# Development of a Blocking Enzyme-Linked Immunosorbent Assay for Detection of Antibodies against African Swine Fever Virus

**DOI:** 10.3390/pathogens10060760

**Published:** 2021-06-17

**Authors:** Fangfeng Yuan, Vlad Petrovan, Luis Gabriel Gimenez-Lirola, Jeffrey J. Zimmerman, Raymond R. R. Rowland, Ying Fang

**Affiliations:** 1 Department of Pathobiology, College of Veterinary Medicine, University of Illinois at Urbana Champaign, Urbana, IL 61802, USA; fy8@illinois.edu; 2 Department of Diagnostic Medicine and Pathobiology, College of Veterinary Medicine, Kansas State University, Manhattan, KS 66506, USA; Vlad.Petrovan@pirbright.ac.uk; 3 Pirbright Institute, Ash Road, Pirbright, Woking, Surrey GU24 0NF, UK; 4 Department of Veterinary Diagnostic & Production Animal Medicine, College of Veterinary Medicine, Iowa State University, Ames, IA 50011, USA; luisggl@iastate.edu (L.G.G.-L.); jjzimm@iastate.edu (J.J.Z.)

**Keywords:** African swine fever virus, ASFV monoclonal antibody, p30, blocking ELISA

## Abstract

The incursion of African swine fever virus (ASFV) into Eurasia presents a threat to the world’s swine industry. Highly sensitive and specific diagnostic assays are urgently needed for rapid detection during an outbreak, post-outbreak investigation, and disease surveillance. In this study, a highly specific and repeatable blocking ELISA (bELISA) was developed using a recombinant p30 protein as the antigen combined with biotinylated mAb against p30 as the detection antibody. Initial test validation included sera from 810 uninfected animals and 106 animals experimentally inoculated with ASFV or recombinant alphavirus/adenovirus expressing p30. Receiver operating characteristic (ROC) analysis of the data calculated an optimal percentage of inhibition (PI) cutoff value of 45.92%, giving a diagnostic sensitivity of 98.11% and diagnostic specificity of 99.42%. The coefficient of variation of an internal quality control serum was 6.81% for between runs, 6.71% for within run, and 6.14% for within plate. A time course study of infected pigs showed that bELISA was able to detect seroconversion as early as 7 days post-inoculation. Taken together, these results demonstrate that bELISA can be used as an alternative serological test for detecting ASFV infection.

## 1. Introduction

African swine fever virus (ASFV) is a large double-stranded DNA virus in the family *Asfarviridae*, genus *Asfivirus* [1]. The virus is enveloped with two membranes at its inner and outer layers, which are wrapped around an icosahedral capsid. The viral genome varies in length (170 to 190 kb), encoding over 170 proteins. In domestic pigs, ASFV infection with virulent isolates causes a lethal hemorrhagic disease, resulting in high morbidity and mortality [2,3]. Pigs that recover from acute infection can go on to sustain a chronic persistent infection [4,5]. The virus initially spread from West Africa to Europe and the Western Hemisphere in the middle of the last century but was eradicated from these areas by the mid-1990s, with the exception of the island of Sardinia, where it remains endemic [6,7,8,9]. Beginning in 2007, ASFV spread from Africa to the Caucasus region and then into Eastern Europe, causing outbreaks in the Russian Federation and several neighboring countries, including Belarus, Ukraine, Lithuania, Estonia, Poland, Latvia, Czech Republic, Romania, and Hungary [10]. By July 2018, 334 outbreaks were reported in Europe. ASFV spread throughout Romania with outbreaks in more than 1000 domestic pig farms in 2018 and about 2500 in 2019 [11]. ASFV first appeared in China in August 2018. As of 18 February 2021, China had reported 187 ASF outbreaks in 31 different administrative divisions according to the World Organization for Animal Health (OIE). During the year of 2020, the Animal Disease Notification System of the European Commission (ADNS) recorded a total of 11,207 ASF outbreaks in wild boar populations in 14 European states (https://www.feedstrategy.com/african-swine-fever/europe-records-fewer-asf-cases-in-pigs-in-2020/ accessed on 7 January 2021). Germany reported its first case on 10 September 2020 (OIE). By 26 October 2020, ASFV outbreaks were reported in more than 50 countries worldwide (https://www.nationalhogfarmer.com/animal-health/fao-oie-kick-initiative-stop-spread-african-swine-fever accessed on 7 January 2021). Even though the US has been free of the virus, ASFV remains a continuous threat.

Currently, there is no effective vaccine available to control ASFV outbreaks. The only effective strategy to control ASF is to quarantine and eliminate infected animals. Therefore, highly sensitive and specific diagnostic assays are critical for rapid detection and isolation of ASFV-infected pigs. After infection, surviving pigs develop a robust antibody response, which can be detected 7 days post-inoculation (DPI) [12,13]. Among all of the ASFV proteins studied so far, p30 is considered the most immunogenic and suitable candidate for antibody detection [14,15]. Amongst different immunoassay platforms, monoclonal antibody (mAb)-based bELISA tests provide a high level of specificity for ASFV antibody detection, e.g., reducing the number of false-positive tests during surveillance of negative populations. In a previous study [16], we produced a panel of mAbs against ASFV p30. One mAb showed specific blocking activity in a bELISA format. In this study, we validated the anti-p30 mAb-based bELISA and applied the test to evaluate the kinetics of antibody response during ASFV infection.

## 2. Results

### 2.1. Antigen Preparation

**Figure 1:**
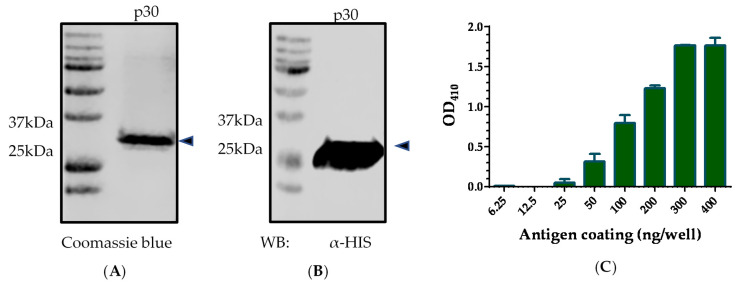
ASFV p30 antigen preparation. (**A**) Sodium dodecyl sulfate–polyacrylamide gel electrophoresis of recombinant p30 protein, followed by Coomassie blue staining. (**B**) Western blot detection of His-tagged p30 protein. The membrane was stained with anti-His tag antibody. Molecular weight marker is shown on the left side. Arrows indicate purified p30 protein. (**C**) Antigen coating amount determined by testing a serially diluted p30 protein in indirect ELISA. *x*-axis represents the amounts of antigens, and *y*-axis shows the OD value.

Synthetic DNA fragment of p30 gene from ASFV BA71V strain was cloned and expressed in *E. coli* as a histidine (His)-tagged recombinant protein. The p30 protein was expressed at a high level but formed inclusion bodies. A protein refolding step was performed after purification. The purity of His-tagged p30 was evaluated in sodium dodecyl sulfate–polyacrylamide gel electrophoresis (SDS-PAGE), followed by Coomassie blue staining. As shown in Figure 1A, Coomassie blue staining showed a sharp band at the predicted size of p30 (~30 kDa) with greater than 90% purity. The identity of the recombinant protein was further confirmed by Western blot analysis using an anti-His mAb (Figure 1B).

The amount of antigen coating on the ELISA plate was determined by two-fold dilution of recombinant p30 protein on a 96-well ELISA plate. Indirect ELISA was performed using the known high-positive serum collected from pigs immunized with alphavirus replicon particles (RP) expressing the p30 (RP-p30) at 71 DPI. The result showed that the coating amount of 300 ng or above generated the highest OD value of 1.8 (Figure 1C).

### 2.2. Establishment of Serum Standards

Internal control standards were established using the sera collected from the terminal bleed (71 DPI) of experimental pigs immunized with alphavirus replicon particles expressing p30 (positive standards) or negative control pigs (negative standards). The positive standards were set as “high-positive”, “medium-positive”, and “low-positive”, with high-positive standard generating an OD of 1.5–2.0, medium-positive standard generating an OD of 1.0–1.5, and low-positive standard generating an OD of 0.8–1.0 in indirect ELISA. The negative standard generated an OD of less than 0.3 in indirect ELISA.

### 2.3. Selection of mAb for Use in p30-Based bELISA

**Figure 2:**
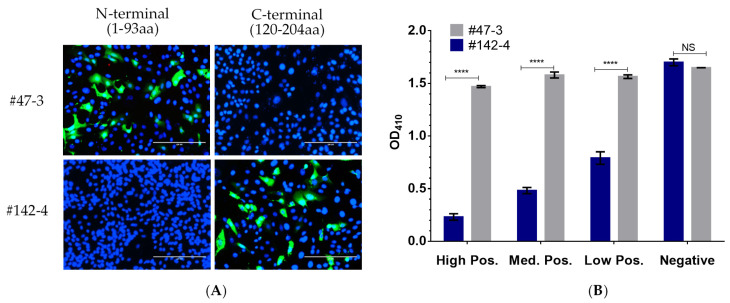
Selection of p30-specific mAb for use in bELISA. (**A**) IFA performed on MARC-145 cells that were transfected with plasmid DNA expressing N-terminal or C-terminal fragment of p30. Cells were fixed and stained with a specific anti-p30 mAb (green) at 48 h post-transfection. Cell nucleus was counter-stained with DAPI (blue). Scale bars, 200 μm. (**B**) Positive and negative control sera were tested to determine the blocking effect for a biotinylated mAb. Each data point is shown as the mean value of two repeats, and the error bar represents the standard deviation. **** *p* < 0.0001; NS, statistically not significant.

In a previous study [16], we produced a panel of mAbs against the ASFV p30 protein. Epitope mapping result showed that anti-p30 mAb #47-3 recognized the epitope [61–93 amino acids (aa)] located in the N-terminal half of p30, while the mAb #142-4 recognized the epitope (120–204 aa) located in the C-terminal region of the p30 protein (Figure 2A). The biotinylated mAbs were tested by bELISA using the internal positive and negative controls. As shown in Figure 2B, the incorporation of mAb #142-4 showed the blocking effect with the positive serum standards, in which the OD value of ELISA was inversely related to the sera antibody concentration. In contrast, mAb #47-3 did not show any blocking effect with the positive serum. Thus, mAb #142-4 was used in the p30-based bELISA development.

### 2.4. Analytical Sensitivity of the p30-Based bELISA

**Figure 3:**
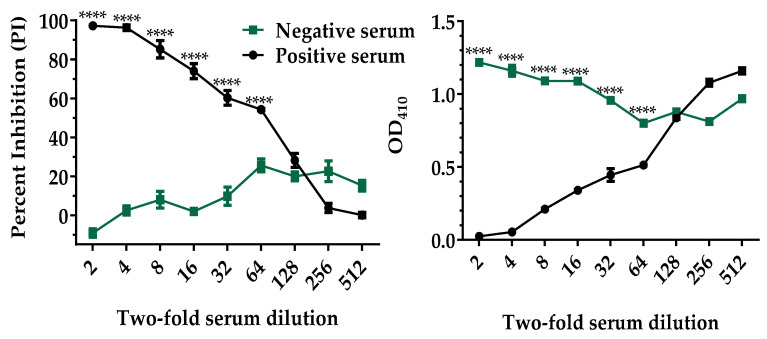
Analytical sensitivity of p30-based bELISA. Two-fold serial dilutions of the high-positive and negative control serum were tested in bELISA. Each data point is shown as the mean value of two repeats, and the error bar represents the standard deviation. Asterisks indicate dilutions at which the PI and OD_410_ of the positive control serum were statistically different from those of negative control serum. **** *p* < 0.0001.

The bELISA conditions were optimized by checkerboard titration of the p30 antigen, mAb, streptavidin conjugate, and other reagents, including blocking buffer and sample dilution buffer. After the assay conditions were optimized, the analytical sensitivity of the p30-based bELISA was evaluated using recombinant p30 antigen, biotinylated mAb#142-4, and the internal control serum standards. High-positive and negative control sera were titrated with two-fold dilutions. As shown in Figure 3, a dilution of 1:64 was the highest dilution producing a statistically difference (*p* < 0.0001) between the positive and negative serum. A 1:4 sample dilution was selected for testing both samples and internal controls as it maximized the discrimination between positive and negative results and minimized background interference.

### 2.5. Diagnostic Sensitivity and Specificity

**Figure 4:**
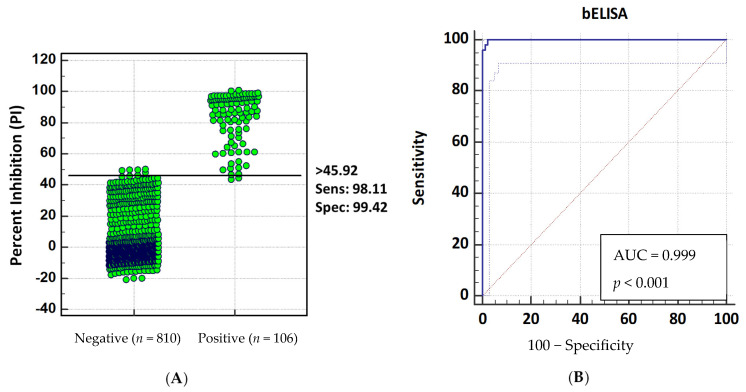
Determination of diagnostic sensitivity and specificity. Receiver operating characteristic (ROC) analysis was performed using 916 serum samples with known ASFV antibody status (810 negatives and 106 positives). (**A**) Interactive plot showing the cutoff value and optimal diagnostic sensitivity and specificity. (**B**) ROC curve showing the accuracy value interpreted as area under the curve (AUC). ROC analysis was performed using MedCalc, version 10.4.0.0 (MedCalc Software, Mariarke, Belgium).

Two sets of serum samples with known antibody status were used to evaluate the diagnostic sensitivity and specificity of the p30-based bELISA. The anti-p30 antibody-positive population consisted of 106 serum samples, including 24 samples collected from pigs infected with ASFV-attenuated OURT 88/3 strain or Georgia/07 strain and 82 samples collected from pigs immunized with recombinant alphavirus or adenovirus particles expressing p30. The anti-p30 antibody-negative population contained 810 serum samples from known ASFV-negative pigs, including 300 samples from Iowa State Veterinary Diagnostic Laboratory (ISU VDL) and 510 samples from experimental pigs in the US. Before testing in bELISA, all samples were analyzed by IFA to confirm the antibody status. The ROC analysis of the bELISA data showed that the cutoff value of 45.92 percent inhibition (PI) produced an optimal diagnostic sensitivity of 98.11% (95% confidence interval of 93.4% to 99.8%) and diagnostic specificity of 99.42% (95% confidence interval of 98.7% to 99.8%.) (Figure 4A). The assay performance was evaluated using a single-graph ROC plot, which was calculated by comparing false-positive results (1 − diagnostic specificity) and true-positive results (diagnostic sensitivity). The area under the curve (AUC) represents the overall accuracy of the assay. An AUC of 1 represents a perfect test, and an AUC above 0.9 indicates high accuracy of the assay. As shown in Figure 4B, the AUC of p30-based bELISA was 0.999 (*p* < 0.001) with a 95% confidence interval of 98.3–100%, demonstrating the high accuracy of the assay.

Based on the selected cutoff value of 45.92%, 5 out of 810 negative serum samples collected from ISU VDL were shown false-positive results, with a PI value ranging from 47.63% to 50.04%, while 2 out of the 106 positive serum samples collected from recombinant alphavirus-vaccinated pigs shown false-negative results with PI values of 44.12% and 43.23%. Of the two false-negative sera samples, the IFA was able to detect antibodies against p30 protein. Similarly, the five false-positive sera samples were confirmed as negative by IFA.

### 2.6. Assessment of bELISA Repeatability

Repeatability measures the ability of a method to generate similar results for multiple preparations of the same sample, while reproducibility determines whether an entire experiment or study can be reproduced. In this study, repeatability and reproducibility were assessed by running a single lot of medium-positive internal control serum. Percent of coefficient of variation (% CV) was calculated as described previously [17]. The result showed that the p30-based bELISA within plate % CV was 6.14% with a mean value of 73.29% and standard deviation of 4.50. The between-plate % CV within one run was 6.71% with a mean value of 73.77% and standard deviation of 4.95. In addition, the % CV between runs was 6.81% with a mean value of 74.01% and standard deviation of 5.04 (Table 1). Regardless, all % CVs were below 10%, indicating that the p30-based bELISA is highly repeatable.

### 2.7. Antibody Response to p30 in ASFV-Infected Pigs

**Figure 5:**
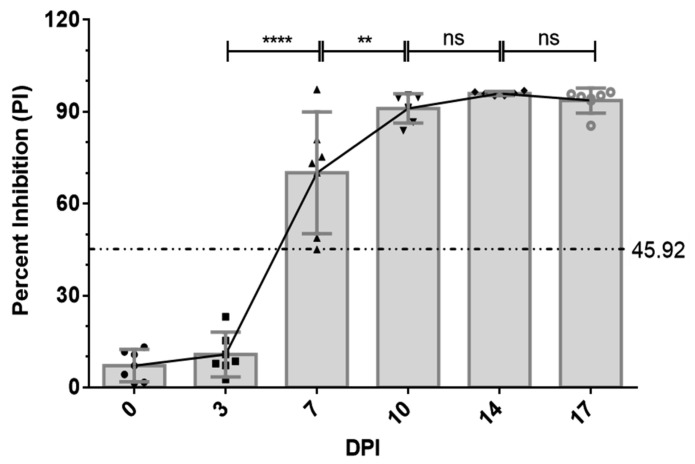
Kinetics of antibody response in serum from ASFV-infected pigs. Serum samples were collected from six pigs infected by ASFV strain OURT 88/3 at 0, 3, 7, 10, 14 and 17 days post-inoculation. The dashed line represents cutoff of bELISA. *x*-axis shows different time points of sample collection. *y*-axis shows calculated percent inhibition. **** *p* < 0.0001; ** *p* < 0.01; ns, statistically not significant.

Next, we applied the p30-based bELISA to determine the humoral immune response in ASFV-infected pigs. A total of six pigs were infected with the attenuated ASFV OURT 88/3 strain. Serum samples were collected twice each week. The ASFV-specific antibody response was determined using bELISA. As shown in Figure 5, the antibody response against p30 protein was detected as early as 7 DPI in five out of six pigs. The p30 response peaked around 10 DPI and remained at a high level through to the end of the experiment (17 DPI).

## 3. Discussion

Recent outbreaks of ASFV in some European and Asian countries pose an increased threat to the global pork industry. Currently, there is no vaccine or other treatments available for ASFV. The principal strategy for control remains early detection, quarantine, and depopulation of affected herds. Cost-effective detection strategies are needed for conducting high-throughput surveillance. Current serological assays for ASF approved by the World Organization for Animal Health (OIE) are based on live virus as the source of antigen, which involves working in high-containment (BSL-3) facilities. This can be overcome using serological assays that utilize noninfectious recombinant ASFV antigens produced in *E. coli* or baculovirus expression systems. There are at least three commercial ELISAs currently available, including the blocking Ingenasa-ELISA (Ingenasa-Ingezim PPA Compac K3; Ingenasa, Madrid, Spain), the indirect IDvet-ELISA (Grabels, France), and the indirect Svanova-ELISA (Svanovir ASFV-Ab; Boehringer Ingelheim Svanova, Uppsala, Sweden). These assays are used as screening tests to confirm ASFV infection status, but they produce a relatively high number of false-positive results. Therefore, effective surveillance may require secondary confirmatory tests, such as the indirect immunoperoxidase test (IPT) [18]. Previous studies have evaluated the performance of different commercial ELISAs, OIE-ELISA, and the immunoperoxidase test [18]. IPT has greater sensitivity than ELISAs, but the test is time-consuming, subjective, and requires working with live virus-derived antigens [18].

Blocking ELISA is generally more specific than the indirect ELISA if an immunodominant epitope is properly employed [17,19]. Although Ingenasa ELISA is a blocking test, it is based on the p72 protein. Previous studies have evaluated common ASFV antigens, including p30, p54, and p72. The results show that p30 elicits the highest level of antibody, which appears relatively early after infection [20,21,22]. In the current study, p30 protein was expressed to high levels in the *E. coli* expression system. To increase the specificity of bELISA, a purified recombinant p30 protein was used as the antigen. As shown by SDS-PAGE, the purity of p30 was greater than 90%. For large-scale preparation, other high-throughput purification methods can be incorporated, such as ion-exchange chromatography, affinity chromatography, and high-pressure liquid chromatography.

To select the specific mAb for incorporating into bELISA, mAb #47-3 and mAb #142-4 were evaluated. In our previous study, the epitope of mAb #47-3 was mapped to the N-terminal region (61–93 aa) of the p30, while epitope mapping of mAb #142-4 showed that the mAb only recognized a large peptide (120–204 aa) at the C-terminal region of the protein [16]. The mAb #142-4 binding epitope covers an intrinsically disordered region possessing a large concentration of serine and glutamic acid residues, which is predicted to be highly immunogenic [16]. This is consistent with the result of a recent animal study, in which the region that conferred immune recognition in ASFV-infected pigs was predominantly located at the C-terminal part of the p30 protein [14]. In the current study, compared to mAb #47-3, mAb #142-4 consistently showed higher PI values with positive control serum. Therefore, mAb #142-4 was selected for use in the development of p30-based bELISA.

The p30 mAb-based bELISA demonstrated good diagnostic sensitivity (98.11%) and specificity (99.42%). With the selected cutoff value of 45.92%, two false-negative and five false-positive results were detected. The PI values for these seven false-negative or false-positive results were close to the cutoff value. The bELISA variation around the cutoff value could be due to several factors, including sample quality and sample storage conditions. It has been reported that serologic testing can be flawed by intrinsic factors, such as autoantibodies [23]. Physical and chemical parameters, such as hemolysis and lipemia, can also affect the test results in the laboratory [24]. In our study, all seven false-positive and false-negative serum samples appeared in dark red color, suggesting they could have been hemolyzed during sample collection.

The p30 mAb-based bELISA was further validated for detecting seroconversion and monitoring the dynamic of antibody response in experimental pigs infected with ASFV strain OURT 88/3. The bELISA was able to detect seroconversion in five out of six pigs at 7 DPI. It detected an increasing trend of antibody response against p30 protein through the time course of the study (0–17 DPI). The detection of specific antibodies at 7 DPI was consistent with the findings in previous studies [12,13]. In comparison to other ELISA settings, indirect ELISA using p30 antigen expressed by baculovirus demonstrates higher sensitivity than that of conventional ELISA that uses whole live virus, but the indirect ELISA can only detect seroconversion at 10 DPI [25]. The capability of our p30 mAb-based bELISA for detection of specific host antibodies at 7 DPI suggests better sensitivity than the other ELISAs for ASFV serological test. Future studies are warranted to conduct field validation of this bELISA in swine populations experiencing ASF outbreaks.

In conclusion, the p30 mAb-based bELISA developed in this study demonstrated a high repeatability with maximized diagnostic sensitivity and specificity in laboratory settings. The assay could be a useful tool for field surveillance and epidemiological studies in swine herd. It also provides an important research tool to study viral pathogenesis/host immunity for development of ASF vaccines.

## 4. Materials and Methods

### 4.1. Production of Recombinant p30 Antigen

Plasmid pHUE containing p30 gene from the ASFV BA71V strain was constructed in a previous study [16]. Recombinant p30 protein was expressed in *E. coli* as a His-tagged fusion protein and purified using Ni–NTA agarose (Qiagen, GmbH, Hilden, Germany). Purified inclusion bodies were further subjected to a protein refolding step using a Novagen protein refolding kit following the manufacturer’s instruction (EMD Biosciences, Darmstadt, Germany). Refolded p30 protein was further dialyzed in 1× phosphate-buffered saline (PBS) three times and concentrated by polyethylene glycol 8000 (Thermo Fisher Scientific, Waltham, MA, USA). The purity of the p30 protein was analyzed by SDS-PAGE. The protein gel was imaged using an Odyssey infrared imaging system (LI-COR Biosciences, Lincoln, NE, USA). Proteins were quantified by measuring the pixel intensity of protein bands using Image Studio 5.2 software (LI-COR Biosciences, Lincoln, NE, USA). The specificity of the protein was confirmed by Western blotting using the anti-His tag monoclonal antibody (HIS.H8) (Thermo Fisher Scientific, Waltham, MA, USA) as described previously [26].

### 4.2. Anti-p30 mAb Production and Biotinylation

A panel of mAbs against the p30 protein was produced in a previous study [16]. Among this panel of antibodies, mAb #142-4 recognizing the C-terminus (120–204 aa) of p30 was selected for bELISA development. The mAb was purified from mouse ascites through ammonium sulfate precipitation and then dialyzed in 1× PBS. Purified mAb #142-4 was used for biotinylation using a biotin conjugation kit (Fast, Type A) (Abcam, Cambridge, MA, USA) following the manufacturer’s instructions. Biotinylated mAbs were aliquoted and stored at −80 °C until use.

### 4.3. Serum Standard and Testing Samples

The internal control sera were collected from pigs immunized with alphavirus replicon particles expressing the p30 [14]. A large quantity of hyperimmune serum from the terminal bleed of RP-p30 immunized pigs at 71 DPI was pooled into a single lot of internal high-positive control serum. Similarly, a large quantity of serum from negative control pigs was pooled into a single lot of internal negative control serum. Medium- and low-positive serum standards were created by spiking high-positive control serum into negative control serum.

Four sets of serum samples were used for bELISA development and validation. The first set of samples (74 samples) was collected at 28, 35, 42, 46, 58, 66, 68, 71 DPI from pigs immunized with RP-p30 [14,15]. The second set of samples (8 samples) was collected from pigs immunized with recombinant adenovirus expressing p30 at 48 DPI [27]. The third set of samples (24 samples) was collected from pigs infected with ASFV genotype I OURT 88/3 strain at 17 DPI (23 samples) and genotype II Georgia/07 strain at 15 DPI (1 sample). These three sets of samples were used to as known positive samples to evaluate the diagnostic sensitivity of the test. The fourth set contained 810 known ASFV-negative serum samples, which were collected from Iowa State Veterinary Diagnostic Laboratory (ISU VDL; 300 samples) and our previous pig experiments (510 samples) in the US [26,28,29]. These samples were used to evaluate diagnostic specificity.

### 4.4. Procedure for ASFV Indirect ELISA and Blocking ELISA

Indirect ELISA was performed using the modified method of our previous study [30]. Briefly, Immulon 2HB plate (Thermo Fisher Scientific, Waltham, MA, USA) was coated with purified p30 antigen at 300 ng/well. Plates were incubated at 37 °C for 1 h, then 4 °C overnight. After blocking with 2% BSA (Sigma-Aldrich, St. Louis, MO, USA), plates were washed three times in wash buffer (0.05% Tween 20 in 1× PBS) and further incubated with serum samples (1:20 dilution in 1% BSA) for 1 h at 37 °C. The plates were washed and incubated with horseradish peroxidase (HRP)-conjugated goat anti-swine IgG (KPL, Gaithersburg, MD, USA). After incubating for 1 h at 37 °C, coloring reactions were developed using ABTS peroxidase substrate (KPL, Gaithersburg, MD, USA). Color development was further quantified at 410 nm with a 96-well BioTek Epoch plate reader (BioTek, Winooski, VT, USA).

The bELISA was designed using swine serum as the primary antibody and a biotinylated anti-p30 mAb as the detection antibody. Anti-p30 antibody presented in positive serum samples binds to the immobilized p30 antigen on the plate, which blocks the binding of secondary biotinylated mAb to the p30 antigen. The mAb is then washed away during the plate-washing step, and no color signal is developed in the following steps. Negative serum samples have no anti-p30 antibody in the serum, and biotinylated mAb therefore binds to immobilized p30 antigen and is then recognized by HRP-conjugated streptavidin for color signal development using peroxidase substrate. Initially, the Immulon 2HB plate (Thermo Fisher Scientific, Waltham, MA, USA) was coated with 100 µL p30 antigen [300 ng of antigen diluted in 100 µL antigen-coating buffer (ACB)] in columns 1, 3, 5, 7, 9, and 11, and the rest columns were coated with antigen-coating buffer. The plate was incubated at 37 °C for 1 h, followed by 16 h incubation at 4 °C. On the next day, the plate was blocked with 200 µL of powerblock (Biogenex Laboratories, Fremont, CA, USA) at 37 °C for 30 min. The plate was washed three times in wash buffer (0.05% Tween 20 in 1× PBS). After washing, 100 µL of the diluted control and testing serum were added and incubated at 37 °C for 1 h. All control and testing serum samples were diluted 1:4 in dilution buffer (0.01% Tween 20 in 1× PBS). Next, 100 µL of biotinylated anti-p30 mAb [1.25 µg mAb diluted in 0.1% BSA (Sigma-Aldrich, St. Louis, MO, USA) in 1× PBS] was added into each well, and the plate was incubated at 37 °C for another 30 min. The plate was washed three times, and 100 µL of streptavidin poly-HRP (Thermo Fisher Scientific, Waltham, MA, USA; 1:2000 dilution in 1% BSA) was then added. After incubation at room temperature for 1 h, the plate was washed six times and subjected to color development using ABTS peroxidase substrate (KPL, Gaithersburg, MD, USA). Reaction was stopped with ABTS peroxidase stop solution (KPL, Gaithersburg, MD, USA) at 10 min after adding the substrate. The plates were read at 410 nm with a 96-well BioTek Epoch plate reader (BioTek, Winooski, VT, USA), and the percent inhibition (PI) was calculated using the following formula:

PI = [1 − (OD of test sample − OD of ACB)/(OD of negative control standard − OD of ACB)] × 100

### 4.5. Immunofluorescent Assay (IFA)

IFA tests on serum samples were performed using a modified procedure as described previously [16]. Vero76 cells (ATCC^®^ CRL-1587™) in 96-well plates were transfected with the pEGFP-C3 vector (Clontech Laboratories, Mountain View, CA, USA) expressing full-length p30. At 48 h post-transfection, cells were fixed with 80% acetone at room temperature for 15 min and then blocked with 2% BSA (Sigma-Aldrich, St. Louis, MO, USA) at 37 °C for 1 h. After washing three times, serum samples (diluted at 1:20 and 1:40 in 1× PBS) were added to the plate and incubated at 37 °C for 1 h. CF594-conjugated goat anti-swine IgG (H + L) (Sigma-Aldrich, St. Louis, MO, USA) was added as the secondary antibody. The plate was washed three times with PBS, and the fluorescent signal was observed under an inverted immunofluorescent microscope LMI6000 (LAXCO, Mill Creek, WA, USA).

To determine epitopes recognized by different mAbs, MARC-145 cells were transfected with p3xFLAG-Myc-CMV-24 vector (SigmaAldrich, St. Louis, MO, USA) expressing N-terminal (1–93 aa) or C-terminal (120–204 aa) fragment of p30. Cells were fixed and stained with a specific anti-p30 mAb (#142-4 or #47-3) at 48 h post-transfection. Alexa-488-conjugated goat anti-mouse IgG (Jackson Immunoresearch, West Grove, PA, USA) was used as the secondary antibody. Cell nucleus was counterstained with 4′,6-diamidino-2-phenylindole (DAPI; Invitrogen, Waltham, MA, USA). The fluorescent signal was observed under an inverted immunofluorescent microscope LMI6000 (LAXCO, Mill Creek, WA, USA).

### 4.6. Validation of bELISA

#### 4.6.1. Analytical Sensitivity Determination

Analytical sensitivity was determined by two-fold serial dilutions of high-positive and negative control serum and tested in triplicate on bELISA. One-way analysis of variance (ANOVA) was performed using GraphPad InStat Prism software to assess differences between different dilutions of the positive and negative control serum.

#### 4.6.2. Cutoff Value, Diagnostic Sensitivity, and Specificity Determination

Test validation was performed on the samples from experimentally immunized/infected pigs described herein. To calculate the optimal cutoff value, and associated diagnostic sensitivity and specificity, serum samples from individual pigs of known ASFV infection/immunization status were tested by bELISA and IFA. Receiver operating characteristic (ROC) analysis was performed to analyze the bELISA results obtained with the positive- and negative-testing sample populations in order to determine an optimized cutoff value that maximizes both diagnostic specificity and diagnostic sensitivity of the assay. The analysis was conducted using MedCalc^®^, version 10.4.0.0 (MedCalc^®^ Software, Mariarke, Belgium).

#### 4.6.3. Repeatability Assessment

The repeatability of bELISA was assessed by running a single lot of internal control serum (medium-positive control). The within-plate assay precision was calculated from 40 replicates on one plate, the within-run assay precision was calculated using a standard serum tested on 10 plates in one run, and the between-run precision was calculated from a standard serum tested in 10 different runs. Means, standard deviations, percent coefficient of variation (% CV), and Levey–Jennings control charts were calculated using Control Chart Pro Plus software (ChemSW, Inc., Fairfield Bay, AR, USA).

#### 4.6.4. Detecting Seroconversion and Antibody Dynamics in ASFV-Infected Pigs

Six Large White x Landrace pigs were housed in the BSL3 animal facility at Kansas Biosecurity Research Institute. The animal experiment was approved by the Institutional Animal Care and Use Committee (IACUC) at Kansas State University. Pigs were inoculated by intramuscular route with 1 × 10^4^ TCID_50_ of low-virulent ASFV OURT 88/3 strain. Serum was collected at 0, 3, 7, 10, 14, and 17 DPI. All collected serum samples were tested by the p30 mAb-based bELISA. Statistical significance of antibody response between the different days was determined by a one-way analysis of variance test using GraphPad InStat Prism software version 6, and a *p*-value of 0.01 (**) was considered as statistically significant.

## Figures and Tables

**Table 1:** Repeatability assessment of p30-based bELISA.

bELISA	Mean (%)	Standard Deviation	Coefficient of Variation ^1^
Within plate	73.29	4.50	6.14
Within run	73.77	4.95	6.71
Between runs	74.01	5.04	6.81

^1^ Values listed are % CVs of a medium-positive internal control serum.

## Data Availability

Data sharing is not applicable to this article.
